# Supplementary material for: Usefulness of Cardiac Computed Tomography in Coronary Risk Prediction: A Five-Year Follow-Up of the SPICA Study (Secure Prevention with Imaging of the Coronary Arteries)
Source: J Clin Med. 2022 Jan 21;11(3):533. doi: 10.3390/jcm11030533 (PMC8836950; doi:10.3390/jcm11030533)
Supplement: Supplementary file 1 [file jcm-11-00533-s001.zip › jcm-1510366-supplementary.pdf]

SUPPLEMENTARY DATA

Table S1. Number of participants by REGICOR risk and CAC categories.

	CAC = 0	CAC > 0	CAC = 1-99	CAC = 100-300	CAC >300
REGICOR CAD risk					
<5	61	17	11	3	3
5-9.9	32	44	21	11	12
≥10	12	62	24	13	25

CAC: Coronary Artery Calcium; CAD: coronary artery disease; REGICOR: *Registre Gironí del Cor/*Girona Heart Registry.

Table S2. Number of participants by REGICOR risk and Segment Involvement Score categories.

	SIS = 0	SIS > 0	SIS = 1-4	SIS = 5-16
REGICOR CAD risk				
<5	59	27	21	6
5-9.9	27	58	37	21
≥10	10	66	30	36

CAD: coronary artery disease; REGICOR: *Registre Gironí del Cor/*Girona Heart Registry; SIS: Segment Involvement Score.

Table S3. Baseline characteristics of the cohort by categories of Agatston Score (CAC).

	All<br>n=216	CAC = 0<br>n=94	CAC = 1-99<br>n=56	CAC = 100-300<br>n=25	CAC > 300<br>n=41	p-trend
Age (years)	58.5 (11.5)	52.6 (12.0)	61.2 (9.17)	62.0 (7.88)	66.3 (7.40)	<0.001
Gender (% female)	59 (27.3%)	43 (45.7%)	11 (19.6%)	2 (8.00%)	3 (7.32%)	<0.001
Total cholesterol (mgr/dL)	200 (38.2)	206 (40.0)	204 (39.0)	198 (33.5)	183 (31.1)	0.002
HDL cholesterol (mgr/dL)	52.3 (16.7)	55.8 (19.9)	51.0 (16.2)	49.3 (10.4)	47.9 (9.48)	0.006
Systolic blood pressure (mm Hg)	133 (17.9)	127 (18.1)	136 (17.5)	140 (16.9)	139 (14.0)	<0.001
Diastolic blood pressure (mm Hg)	82.5 (10.7)	81.0 (11.8)	83.5 (12.0)	85.0 (7.34)	83.2 (7.61)	0.143
Diabetes (%)	52 (24.4%)	15 (16.1%)	13 (24.1%)	5 (20.0%)	19 (46.3%)	0.001
Smoking (%)	64 (29.6%)	26 (27.7%)	13 (23.2%)	12 (48.0%)	13 (31.7%)	0.289
REGICOR CAD risk	13.0 (7.52)	9.04 (6.78)	15.1 (7.15)	15.0 (5.04)	17.7 (6.74)	<0.001
CAC (Arbitrary units)	220 (605)	0.00 (0.00)	30.9 (27.6)	186 (61.0)	1003 (1081)	<0.001
SIS (number of segments with plaque)	2.73 (3.20)	0.22 (0.64)	2.39 (1.70)	5.16 (1.86)	7.44 (2.60)	<0.001
Percent SIS <5	159 (73.6%)	94 (100%)	50 (89.3%)	9 (36.0%)	6 (14.6%)	<0.001
CAD events in the follow-up (%)	9 (4.17%)	0 (0.00%)	3 (5.36%)	2 (8.00%)	4 (9.76%)	0.005

Data are shown as mean ± standard deviation except for gender, diabetes, smoking, individuals with CAC ≤0, and individuals with Segment Involvement Score (SIS) < 5 (between 0 and 4), for which the number of individuals and the percentage is presented. AS: Agatston Score; CAD: coronary artery disease; HDL: high-density lipoprotein cholesterol; HU: Hounsfield units; REGICOR: *Registre Gironí del Cor/*Girona Heart Registry.

Table S4. Baseline characteristics of the cohort by categories of Segment Involvement Score.

	All<br>n=233	SIS = 0<br>n=85	SIS = 1-4<br>n=85	SIS > 4<br>n=63	p-trend
Age (years)	58.3 (11.3)	51.8 (12.0)	60.2 (9.12)	64.5 (8.23)	<0.001
Gender (% female)	59 (25.3%)	39 (45.9%)	17 (20.0%)	3 (4.76%)	<0.001
Total cholesterol (mgr/dL)	201 (39.3)	206 (40.8)	205 (35.9)	189 (39.6)	0.010
HDL cholesterol (mgr/dL)	53.0 (17.6)	56.3 (20.6)	53.5 (18.1)	48.0 (10.3)	0.005
Systolic blood pressure (mm Hg)	133 (17.5)	126 (17.0)	135 (16.1)	140 (16.6)	<0.001
Diastolic blood pressure (mm Hg)	82.6 (10.6)	80.2 (11.8)	83.5 (10.3)	84.5 (8.95)	0.013
Diabetes (%)	53 (23.0%)	12 (14.3%)	17 (20.5%)	24 (38.1%)	0.001
Smoking (%)	68 (29.2%)	21 (24.7%)	23 (27.1%)	24 (38.1%)	0.087
REGICOR risk	12.7 (7.63)	8.45 (6.05)	13.4 (7.03)	17.4 (7.32)	<0.001
CAC (Arbitrary units)	220 (605)	0.02 (0.22)	73.4 (125)	735 (1006)	<0.001
Percent CAC = 0	94 (43.5%)	82 (98.8%)	12 (15.8%)	0 (0.00%)	<0.001
SIS (number of segments with plaque)	2.79 (3.17)	0.00 (0.00)	2.26 (1.11)	7.29 (2.10)	<0.001
CAD events in the follow-up (%)	9 (3.86%)	0 (0.00%)	1 (1.18%)	8 (12.7%)	<0.001

Data are shown as mean ± standard deviation except for gender, diabetes, smoking, individuals with CAC ≤0, and individuals with Segment Involvement Score (SIS) between 0 and 4, for which the number of individuals and the percentage is presented. CAD: coronary artery disease; HDL: high-density lipoprotein cholesterol; REGICOR: *Registre Gironí del Cor/*Girona Heart Registry; SIS: Segment Involvement Score.
